# Social media attention and citations of published outputs from re-use of clinical trial data: a matched comparison with articles published in the same journals

**DOI:** 10.1186/s12874-021-01311-z

**Published:** 2021-06-06

**Authors:** N. Anthony, C. Pellen, C. Ohmann, D. Moher, F. Naudet

**Affiliations:** 1 University Hospital of La Réunion, Saint-Denis, Reunion Island France; 2 Univ Rennes, CHU Rennes, Inserm, CIC 1414 [(Centre d’Investigation Clinique de Rennes)], F-35000 Rennes, France; 3 European Clinical Research Infrastructure Network, Düsseldorf, Germany; 4 Ottawa Hospital Research Institute, Ottawa, Canada

**Keywords:** Data-sharing, Data reuse, Altmetric, Individual Participant Data, Clinical trial, Scientific transparency, Reproducibility, Attention score, Research impact

## Abstract

**Background:**

Data-sharing policies in randomized clinical trials (RCTs) should have an evaluation component. The main objective of this case–control study was to assess the impact of published re-uses of RCT data in terms of media attention (Altmetric) and citation rates.

**Methods:**

Re-uses of RCT data published up to December 2019 (cases) were searched for by two reviewers on 3 repositories (CSDR, YODA project, and Vivli) and matched to control papers published in the same journal. The Altmetric Attention Score (primary outcome), components of this score (e.g. mention of policy sources, media attention) and the total number of citations were compared between these two groups.

**Results:**

89 re-uses were identified: 48 (53.9%) secondary analyses, 34 (38.2%) meta-analyses, 4 (4.5%) methodological analyses and 3 (3.4%) re-analyses. The median (interquartile range) Altmetric Attention Scores were 5.9 (1.3—22.2) for re-use and 2.8 (0.3—12.3) for controls (*p* = 0.14). No statistical difference was found on any of the components of in the Altmetric Attention Score. The median (interquartile range) numbers of citations were 3 (1—8) for reuses and 4 (1 – 11.5) for controls (*p* = 0.30). Only 6/89 re-uses (6.7%) were cited in a policy source.

**Conclusions:**

Using all available re-uses of RCT data to date from major data repositories, we were not able to demonstrate that re-uses attracted more attention than a matched sample of studies published in the same journals. Small average differences are still possible, as the sample size was limited. However matching choices have some limitations so results should be interpreted very cautiously. Also, citations by policy sources for re-uses were rare.

**Trial registration:**

Registration: osf.io/fp62e

**Supplementary Information:**

The online version contains supplementary material available at 10.1186/s12874-021-01311-z.

## Key messages

- Few re-uses have been published since the creation of the three data-sharing platforms.
- Most of them were secondary analyses or meta-analyses.
- No systematic impact of re-use was found in terms of the Altmetric Attention Score, media attention, social media attention and citation metrics however small averaged differences in favor of re-use are still plausible.
- Citations by policy sources of reuses were rare, and likewise for the controls.
- Re-uses with highest Altmetric Attention Score were mainly IPD meta-analyses
- Re-analyses were rare, however two out of the three were among the re-uses with the highest Altmetric Attention Score.
- Importantly, a high Altmetric Attention Score does not imply high scientific quality.

## Introduction

In recent years, a cultural shift in research practices has occurred with the promotion of clinical trial data-sharing and reuse [1, 2]. An increasing number of journals tend to have a data-sharing policy and encourage researchers to publish their data. Both the BMJ and PLOS Medicine even require data-sharing as a condition for publication of randomized controlled trials (RCTs). This change has taken place in a favorable scientific context. The growing movement within the scientific community to make science more open and accessible [3], as well as the awareness of a reproducibility crisis [4], are contributing to the adoption of more transparent research practices [5] that include data-sharing. Recent initiatives such as the FAIR guidelines (findable, accessible, interoperable and reusable) provide guidance on how to improve the reusability of data [1].

Sharing data generated by interventional clinical trials is considered an ethical obligation according to the International Committee of Medical Journal Editors (ICMJE) [6]. As participants have put themselves at risk in clinical trials, it is a moral obligation that the value arising from the collected data should be maximized. Besides enhancing both reproducibility and transparency, data-sharing optimizes the use of limited resources (time, costs and number of patients exposed) hence reducing research wastage [7–10]. In terms of improvement of scientific knowledge, clinical trial data can set the basis for the generation of biomedical hypotheses and even hypothesis validation through the meta-analysis of Individual Participant Data (IPD). Finally, the accumulation of unbiased data on the safety and efficacy of the therapeutics being tested is crucial to better inform public health decisions, and indeed to improve the care that is offered to patients [11, 12].

However, this new paradigm faces barriers related to the feasibility of the requirements, the resources required, and the potential threats to trial participants [13]. Indeed, clinical trial data requires specific precautions because of the management of sensitive data, and it entails an additional workload for data-managers. Usually the sponsors are the data managers of the clinical trial data. The attitude and role of sponsors towards data-sharing can also contribute to reluctance. There is no standardization of policies across funders of clinical trials, and for those with a policy, implementation was sub-optimal [14]. Other barriers such as “fear of being wrong in public and concerns about so-called hostile re-analysis” can also limit acceptance of data-sharing practices [15].

To address these barriers, data-sharing policies should have an evaluation component to demonstrate whether the benefits outweigh the possible shortcomings. This evaluation component is currently lacking. To date, there has been no systematic attempt to assess the impact of published re-uses from clinical trials which we expected to be important, in line with the expectations of the ICMJE [6]. In a first step, an analysis of the output from data repositories can provide some useful information. These repositories, such as the YODA project [16], Clinical Study Data Request (CSDR) [17] and VIVLI [18], enable controlled access to and re-use of anonymized IPD from clinical trials. The main objective of this case–control study was therefore to assess the impact of published re-uses of data from clinical trials in terms of media attention (Altmetric) and citation rates.

## Method

The protocol of the study was registered on Open Science Framework (OSF) on December 27th 2019 (registration number osf.io/fp62e). As there are no specific guidelines for meta-research studies, we have adapted the PRISMA checklist [19] as a guide to report our study.

### Eligibility criteria

#### Cases (Re-uses)

All published re-uses on 3 data repositories were assessed: clinicalstudydatarequest.com (CSDR), Yale University Open Data Access (YODA), and Vivli. These data repositories were chosen because they were identified as the 3 main data repositories in a previous scoping review [20]. CSDR, funded by a consortium of clinical funders/sponsors, seeks to be “the researcher-preferred and trusted” platform for responsible sharing of high-quality patient-level data for the purpose of facilitating innovative data-driven research leading to improvements in patient care. As of 31 January 2020 there was 2123 RCTs listed. The YODA project, funded by Johnson & Johnson (formerly funded by Medtronic Inc), “advocates the responsible sharing of clinical research data, open science, and research transparency”. The number of RCTs listed was 334 in July 2019. Vivli runs a data-sharing platform that includes an independent-access general data repository which is available to search for data from clinical trials conducted by researchers in academic institutions, industry, foundations and non-profit entities that can be hosted, shared and accessed. The number of RCTs listed was 4920 in March 2020. These 3 repositories, as part of their metrics, detail all published outputs related to the randomized clinical trial (RCT) data that they host [20]. Any re-use mentioned on these platforms and published in a peer review journal in English, French, German or Spanish between the creation of the platforms and 31^st^ December 2019 was considered. Re-uses published only as part of congress proceedings were not included nor were re-uses not listed on PubMed. The re-uses were classified as secondary analyses, meta-analyses, methodological analyses or re-analyses.

#### Controls

Each selected publication of a re-use was matched with an article published in the same journal. In case of re-uses published in specialty journals, the article listed immediately before or after the re-use in the table of contents was randomly chosen. In the absence of journals published by volume/issue (therefore absence of table of contents), online publication dates were used to choose the closest paper published before or after in the same journal. In case of re-uses published in a general medical journal, matching was performed by research field (using the International Classification of Diseases categorization: ICD-11 for Mortality and Morbidity Statistics (ICD-11 MMS), 2018 version https://icd.who.int/browse11/l-m/en), and the closest control with matching content, randomly chosen between before and after and based on online publication date, was selected. RCT data re-uses or publications on non-human data were not considered for the formation of the control group. All controls were classified according to whether or not they were clinical trials.

#### Primary publications

A "primary publication" group was also set up, including publications from which the re-uses were derived. For a given re-used RCT, a “primary publication” (i.e. the main publication of the dataset that was re-used) was defined as the first article reporting fully the primary outcome of the study. In many cases (e.g. IPD meta-analyses), there were several primary publications per re-use. Also, some primary publications were related to several different re-uses. These primary publications were sought on the data-sharing platform, the clinical trial register (e.g. clinicaltrials.gov) and the manufacturer website.

#### Data sources

Two reviewers (NA and CP) among a team of four (NA, CP, FN, CO) independently assessed the 3 platforms. PMID (PubMed identifier) were used as identifiers for re-uses. In case of disagreement between the pair of independent reviewers, a third reviewer arbitrated. The matching of re-uses with controls and the identification of primary publications was performed by one reviewer (NA).

### Outcomes

#### Primary outcome

The primary outcome was the Altmetric Attention Score. This score is an automatically calculated, weighted count of all of the mentions the Altmetric company has tracked for an individual research output, and is designed as an indicator of the amount and the extent of the attention an item has received [21]. The Altmetric Attention Score is mostly based on social media attention but also includes attention in newspapers and in policy sources. It is an increasingly recognized tool, which aims to measure the real-time influence and outreach of academic articles [22].

#### Secondary outcomes

The secondary outcomes were the citation metrics (total citations) and the following components of the Altmetric Attention Score: 1/ Policy sources (number of policy sources citing the paper), 2/ News (number of media sources that have mentioned the publication), 3/ Blogs (number of blogs that have mentioned the publication), 4/Twitter (number of the twitter accounts that have tweeted the publication), 5/ Facebook (number of the pages that have been shared on Facebook), 6/ Reddit (number of Reddit threads posted about this publication), 7/ Video (number of Youtube/Vimeo videos), 8/ Google plus (Number of accounts that have shared on Google +), 9/ Readers (Total Reader Counts, readers in citeulike count, readers in Mendeley count, readers in Connotea count).

The primary outcome and the citation metrics were assessed for the three groups (re-uses, controls, and primary publications) and the remaining secondary outcomes were assessed for re-uses and controls only. In the case of multiple primary publications for a re-use, the average of the quantitative outcomes among these publications was used.

### Data analysis

We first performed a descriptive analysis of the general characteristics of cases and controls using basic statistical parameters (medians, first and third quartile for quantitative variables, counts and percentages for qualitative variables).

Then, the re-use and control groups were compared using the Wilcoxon rank sum test on all primary and secondary outcomes. We relied on non-parametric analyses, because of the highly skewed distribution of these outcomes, especially the Altmetric Attention Score.

As part of a secondary analysis, correlations between the Altmetric Attention Scores of the primary publications and those for re-uses, and between the number of citations of primary publications and re-uses, were explored and tested using the Spearman test. Lastly, an exploratory analysis determined whether certain types of content (Medicine (adults and children) and psychology/psychiatry (adults and children) were associated with more attention or higher citation rates. For these two groups of analysis, an adjustment by Bonferroni was performed following a comment in the peer review process.

Altmetric score-related outcomes were automatically extracted from Altmetric explorer using the R library (rAltmetric) [23]. We used the 3.4.2 version of R [24]. rAltmetric retrieves data from Altmetric explorer by means of an API (application programming interface) key [25]. Agreements for the use of Altmetric data using an API key were reached with the Altmetric team on the 5th December 2019. Selection and coding were performed by four independent reviewers in independent manner.

## Results

### Changes to the initial protocol

In order to provide a qualitative appraisal of the impact of re-use, we added a description of the 20 re-uses that attracted the most attention and the detail and context for all re-uses cited by a policy source. While we planned that two researchers would review the primary studies, only one did, given the secondary nature of the analysis and the unfavorable ratio of time spent to informativeness. Finally, two additional secondary outcomes, “score in context rank within publications in the journal” and “score in context rank within publications in this time period” were planned but not analyzed because we were no able to extract the overall number of ranks data making their interpretation impossible.

### Study identification

**Fig. 1:**
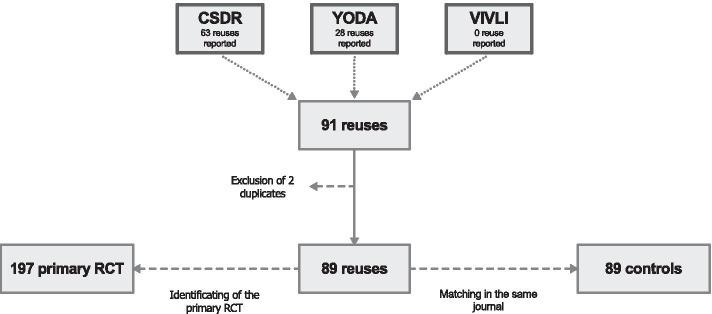
Flow chart of study selection. The following figure was represented in a square root scale

Matching and data extraction occurred in February 2020. Data on Altmetric metrics and the number of citations were extracted on the 10th of April 2020, as part of the final analysis. Sixty-three re-uses were extracted from CSDR and 28 from YODA. There were no re-uses found on the VIVLI repository. After deletion of duplicates and matching, a total of 89 re-uses (with 197 primary RCTs identified) and 89 matched controls was obtained (Fig. 1).

### Description of studies included

Re-uses from the two repositories published since 2015 were as follows 48 (53.9%) of them were secondary analyses (7 were from YODA and 41 from CSDR), 34 (38.2%) were meta-analyses (19 were from YODA and 15 from CSDR), 4 (4.5%) methodological analyses (one was from YODA and 3 from CSDR) and 3 (3.4%) re-analyses (one was from YODA and 2 from CSDR). For the controls, 76 (85.4%) were not clinical trials and 13 (14.6%) were clinical trials (Web appendix 1). A total of 66 (74.2%) re-uses were in the area of adult medicine, 18 (20.2%) on psychology/psychiatry (children and adults) and 5 (5.6%) on pediatrics. A total of 8 of 89 reuses were from general medical journals and required matching by ICD classification (Web appendix 2).

### Media attention and impact of re-use

**Fig. 2:**
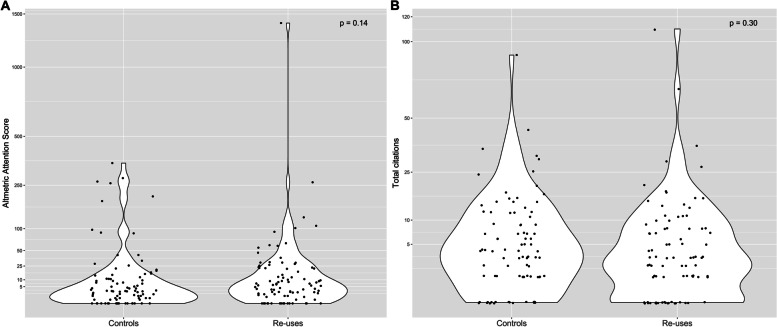
Outcome distributions and comparisons between re-uses and controls. **a**. Altmetric Attention Score. **b**. Total citations

The median (interquartile range) Altmetric Attention Scores (Fig. 2a) were 5.9 (1.3–22.2) for re-uses and 2.8 (0.3–12.3) for controls (*p* = 0.14). No statistical difference was found on the various components of the Altmetric Attention Score (Table1). Median (interquartile range) numbers of citations (Fig. 2b) were 3.0 (1.0–8.0) for reuses and 4 (1.0–11.5) for controls (*p* = 0.30). Detail per article type is provided in Web appendix 3. The median (1^st^ quartile, third quartile) lag between re-uses and controls was 0 (0, 0).

**Table 1:** Altmetric data for reuses and controls

	Control (*N* = 89)	Re-use (*N* = 89)	Total (*N* = 178)	***p***-value
Altmetric Attention Score	2.8 (0.3—12.3)	5.9 (1.3—22.2)	3.9 (0.5—15.9)	0.14
Number of citations^a^	4 (1 – 11.5)	3 (1—8)	3 (1—9)	0.30
Number of policy sources that have mentioned the publication	0 (0—0)	0 (0—0)	0 (0—0)	0.52
Number of the news sources that have mentioned the publication	0 (0—0)	0 (0—1)	0 (0 – 0.8)	0.64
Number of blogs that have mentioned the publication	0 (0—0)	0 (0—0)	0 (0—0)	0.17
Number of the Twitter accounts that have tweeted this publication	4 (1 – 10)	5 (1 – 17)	4 (1 – 13.75)	0.14
Number of pages shared on Facebook	0 (0—1)	0 (0—1)	0 (0—1)	0.74
Number of Reddit threads posted about this publication	0 (0—0)	0 (0—0)	0 (0—0)	0.17
Number of the Youtube/Vimeo channels	0 (0—0)	0 (0—0)	0 (0—0)	1
Number of the accounts that have shared on Google +	0 (0—0)	0 (0—0)	0 (0—0)	0.40
Total Reader Counts	15 (2—36)	15 (4—31)	15 (3.3 – 35.3)	0.91
Readers in citeulike	0 (0—0)	0 (0—0)	0 (0—0)	0.32
Readers in Mendeley	15 (2—36)	15 (4—31)	15 (3.3 – 35.3)	0.90
Readers in Connotea	0 (0—0)	0 (0—0)	0 (0—0)	/

For all quantitative outcomes, data are presented as median (interquartile range)

^a^ 5 missing data (2 for controls and 3 for re-uses)

A total of 6 (6.7%) re-uses were mentioned by a policy source (Table 2): 2 meta-analyses [26, 27], 3 secondary analyses [28–30] and one re-analysis [31]. One meta-analysis and one secondary analysis were cited in “immunological basis for immunization series” of the World Health Organization (WHO). The other meta-analysis was cited in the National Institute for Health Care Excellence (NICE) guidelines as a reference for psoriatic arthritis following inadequate response to disease-modifying anti-rheumatic drugs, and its economic evaluation. Another secondary analysis was identified by Altmetric as cited by a NICE evidence review, but after careful evaluation of the NICE reviews, it appeared that this study was cited as an excluded study and therefore had no practical impact. The last secondary analysis was cited in a national immunisation programme in the Netherlands. The re-analysis was cited as an example demonstrating the need for Open Data.

**Table 2:** Re-uses cited by policy sources

Published outputs	Title	Type of study	Policy sources	How cited
Joanna Le Noury et al. 2015	Restoring Study 329: efficacy and harms of paroxetine and imipramine in treatment of major depression in adolescence	Re-analysis	The state of open data. 25 Oct 2016 https://doi.org/10.6084/m9.figshare.4036398	In a chapter stating the ethical necessity of open data in medical research, the re-use is cited as a subsequent analysis that debunked the previous results of the study 329 which stated that “Paroxetine is generally well tolerated and effective for major depression in adolescents”
Merryn Voysey et al. 2017	The Influence of Maternally Derived Antibody and Infant Age at Vaccination on Infant Vaccine Responses	Meta-analysis	World Health Organization. (2018). The immunological basis for immunization series: module 3: tetanus: update 2018. World Health Organization. https://apps.who.int/iris/handle/10665/275340. License: CC BY-NC-SA 3.0 IGO	The re-use was cited in the immunological basis for immunization series: module 3: tetanus: update 2018 from World Health Organization but the exact context in the text was not found
Joseline Guetsop Zafack et al. 2019	Adverse events following immunisation with four-component meningococcal serogroup B vaccine (4CMenB): interaction with co-administration of routine infant vaccines and risk of recurrence in European randomised controlled trials	Secondary analysis	The National Immunisation Programme in the Netherlands: Surveillance and developments in 2018–2019	The re-use was cited in the national immunisation programme in the Netherlands for the 2018–2019 period but the exact context was not found in the text
Mark Corbett et al. 2017	Certolizumab pegol and secukinumab for treating active psoriatic arthritis following inadequate response to disease-modifying antirheumatic drugs: a systematic review and economic evaluation	Meta-analysis	National Institute for Health Care Excellence (NICE) guidance: Ixekizumab for treating active psoriatic arthritis after inadequate response to DMARDs (https://www.nice.org.uk/guidance/ta537/evidence/committee-papers-pdf-4913117821)	The meta-analysis was cited in NICE as a reference regarding the use of a York model and details on its implementation (5 times): a reference regarding resource use estimates for controlled and uncontrolled psoriasis, a reference for psoriasis severity classification according to the York model, a reference for psoriasis evolution without treatment and a reference stating the lack of clinically meaningful difference in bDMARD responses rates for joint disease or psoriasis between 12 to 24 weeks
G. A. Mospan et al. 2016	5-Day versus 10-Day Course of Fluoroquinolones in Outpatient Males with a Urinary Tract Infection (UTI)	Secondary analysis	NICE guidance: Urinary tract infection (lower): antimicrobial prescribing (evidence review)	The re-use is cited as an excluded study in the NICE evidence review (non-relevant population) so it did not contribute to the policy source content
Lauren M. Schwartz et al. 2016	Rotavirus vaccine effectiveness in low-income settings: An evaluation of the test-negative design	Secondary analysis	World Health Organization. (2020). The immunological basis for immunization series: module 21: rotavirus vaccines. World Health Organization. https://apps.who.int/iris/handle/10665/331323. License: CC BY-NC-SA 3.0 IGO	The re-use was cited in the immunological basis for immunization series: module 21: rotavirus vaccines from World Health Organization but the exact context was not found in the text

The top 20 re-uses with the highest Altmetric Attention Scores ranging from 23.5 to 1407.5 are presented in Table 3: 12 (60%) were in fields of medicine, 7 (35%) in psychology/psychiatry and one in pediatrics (5%). Among re-uses, 11 (55%) were IPD meta-analyses, 5 (25%) were secondary analyses, 2 (10%) were methodological papers and 2 (10%) were re-analyses.

**Table 3:** Top 20 re-uses with highest Altmetric Attention Scores

Published outputs	Title	Theme	Platform	Type of study	Altmetric score	Tweets	Policy sources	Newspapers
Joanna Le Noury et al. 2015	Restoring Study 329: efficacy and harms of paroxetine and imipramine in treatment of major depression in adolescence	Psychology/psychiatry (child and adults)	CSDR	Re-analysis	1407.5	X	X	X
F Hieronymus et al. 2017	Efficacy of selective serotonin reuptake inhibitors in the absence of side effects: a mega-analysis of citalopram and paroxetine in adult depression	Psychology/psychiatry (child and adults)	CSDR	Meta-analysis	262.6	X		X
Johannes Schneider-Thoma et al. 2018	Second-generation antipsychotic drugs and short-term mortality: a systematic review and meta-analysis of placebo-controlled randomised controlled trials	Psychology/psychiatry (child and adults)	YODA	Meta-analysis	132.7	X		X
Johannes Schneider-Thoma et al. 2019	Second-generation antipsychotic drugs and short-term somatic serious adverse events: a systematic review and meta-analysis	Psychology/psychiatry (child and adults)	YODA	Meta-analysis	107.9	X		X
Monique B. Nilsson et al. 2017	Stress hormones promote EGFR inhibitor resistance in NSCLC: Implications for combinations with ß-blockers	Medicine (adults)	CSDR	Secondary analysis	101.6	X		X
John M. Dennis et al. 2018	Sex and BMI Alter the Benefits and Risks of Sulfonylureas and Thiazolidinediones in Type 2 Diabetes: A Framework for Evaluating Stratification Using Routine Clinical and Individual Trial Data	Medicine (adults)	CSDR	Secondary analysis	91.8	X		X
Sarah J Nevitt et al. 2017	Exploring changes over time and characteristics associated with data retrieval across individual participant data meta-analyses: systematic review	Medicine (adults)	CSDR	Methodological	64.9	X		
Siddharth Singh et al. 2018	No Benefit of Concomitant 5-Aminosalicylates in Patients With Ulcerative Colitis Escalated to Biologic Therapy: Pooled Analysis of Individual Participant Data From Clinical Trials	Medicine (adults)	YODA	Meta-analysis	60.7	X		X
Akbar K. Waljee et al. 2019	Development and Validation of Machine Learning Models in Prediction of Remission in Patients With Moderate to Severe Crohn Disease	Medicine (adults)	YODA	Secondary analysis	59.4	X		X
Z.Z.N. Yiu et al. 2019	A standardization approach to compare treatment safety and effectiveness outcomes between clinical trials and real-world populations in psoriasis	Medicine (adults)	YODA	Methodological	55.6	X		X
Hawkins C. Gay et al. 2017	Feasibility, Process, and Outcomes of Cardiovascular Clinical Trial Data Sharing	Medicine (adults)	YODA	Re-analysis	46	X		X
Merryn Voysey et al. 2017	The Influence of Maternally Derived Antibody and Infant Age at Vaccination on Infant Vaccine Responses	Paediatric	CSDR	Meta-analysis	37.3	X	X	X
Ymkje Anna de Vries et al. 2018	Predicting antidepressant response by monitoring early improvement of individual symptoms of depression: individual patient data meta-analysis	Psychology/psychiatry (child and adults)	CSDR	Meta-analysis	30.4	X		
Shereen Oon et al. 2019	Lupus Low Disease Activity State (LLDAS) discriminates responders in the BLISS-52 and BLISS-76 phase III trials of belimumab in systemic lupus erythematosus	Medicine (adults)	CSDR	Secondary analysis	29.9	X		X
Ymkje Anna Vries et al. 2018	Initial severity and antidepressant efficacy for anxiety disorders, obsessive–compulsive disorder, and posttraumatic stress disorder: An individual patient data meta-analysis	Psychology/psychiatry (child and adults)	CSDR	Meta-analysis	29.8	X		
Joseline Guetsop Zafack et al. 2019	Adverse events following immunisation with four-component meningococcal serogroup B vaccine (4CMenB): interaction with co-administration of routine infant vaccines and risk of recurrence in European randomised controlled trials	Medicine (adults)	CSDR	Secondary analysis	29.6	X	X	
Andriy Noshchenko et al. 2015	What Is the Clinical Relevance of Radiographic Nonunion After Single-Level Lumbar Interbody Arthrodesis in Degenerative Disc Disease?	Medicine (adults)	YODA	Meta-analysis	27.9	X		X
Runsheng Wang et al. 2018	Comparative Efficacy of Tumor Necrosis Factor-a Inhibitors in Ankylosing Spondylitis: A Systematic Review and Bayesian Network Metaanalysis	Medicine (adults)	YODA	Meta-analysis	26.6	X		X
Jacob Spertus et al. 2018	Risk of weight gain for specific antipsychotic drugs: a meta-analysis	Psychology/psychiatry (child and adults)	YODA	Meta-analysis	25.6	X		X
Amber L. Laurie et al. 2016	Meta-analysis of the Impact of Patient Characteristics on Estimates of Effectiveness and Harms of Recombinant Human Bone Morphogenetic Protein-2 in Lumbar Spinal Fusion	Medicine (adults)	YODA	Meta-analysis	23.5	X		X

A significant negative correlation between primary studies and re-uses for the Altmetric Attention Score was found (*ρ* = -0.31, *p* = 0.006, Web appendix 4) and no correlation was found for citations rates (*ρ* = -0.20, *p* = 0.08, Web appendix 4). Psychiatry/psychology was associated with a higher Altmetric Attention Score than medicine (8.4 (2.3–30.9) for psychiatry/psychology and 3.2 (0.3–12.1) for medicine; *p *= 0.003). Total citations were not found to differ between these two subgroups (5 (1.8–13.3) for psychiatry/psychology and 3 (1.0–8.8) for medicine; *p* = 0.11). There was no difference in terms of Altmetric Attention Scores nor for citations in the subgroups of Psychiatry/psychology (respectively *p* = 0.40 and *p* = 0.69) or in medicine (respectively *p* = 0.24 and *p* = 0.38). Using Bonferroni’s method for multiple comparisons, both the correlation between primary studies and re-uses for the Altmetric Attention Score and the association between psychiatry/psychology content and higher Altmetric Attention Score remained significant over the 8 former correlations/comparisons (*p* < 0.0065). We did not test any interaction because the data was highly skewed and precluded the use of parametric models. All these analyses are detailed in Web Appendix 5.

## Discussion

### Statement of principal findings

Using all available re-uses of clinical trial data to date from two major data repositories, we were not able to demonstrate that re-uses of clinical trial data attracted more attention than a matched sample of studies published in the same journals: while the Altmetric Attention Score was numerically higher for re-uses than for controls, the difference did not reach statistical significance, perhaps because of lack of power. The magnitude of the difference was nevertheless quantitatively small. It is important to stress that whether for re-uses or for controls, a large proportion of articles had a null or low Altmetric Attention Score. In addition, a large part of the Altmetric Attention Score was driven by activity on Twitter and only six out of 89 re-uses were cited in policy sources, with 5 out of these 6 having directly impacted these documents. Unsurprisingly most of the re-uses that attracted attention were IPD meta-analyses, as these studies present in general high-quality evidence and can attract more interest. Interestingly, 2 of the 3 re-analyses were among the re-uses that attracted the most attention. The first one [31] was the first study within the RIAT initiative (Restoring Invisible and Abandoned Trials) [32] which contradicted a previous, well known, misreported trial on paroxetine among adolescents with major depressive disorder. The second one [33] set out to reproduce SMART-AF [34], a multicenter, single-arm trial evaluating the effectiveness and safety of an irrigated, contact force-sensing catheter for ablation of drug-refractory, symptomatic, paroxysmal atrial fibrillation. This re-analysis arrived at the same general conclusions as the initial study. Attention towards re-analyses can be expected, since reproduction analyses are an important outcome from data-sharing. This being said, re-analyses were not commonly performed in the area of medicine [35] and were indeed a small portion of the re-uses we identified.

### Findings in relation to other studies

Up to the present day, evidence on the impact of research output from sharing IPD from clinical trials is still very sparse. In an assessment of citation metrics for 224 re-uses of clinical trial data from the NHLBI Data Repository, half of the publications based on clinical trial data had cumulative citations that ranked in the top 34%, normalized for subject matter and year of publication. No significant difference was found compared to the percentage of 28.3% for publications based on observational studies and 29% for a 10%-random sample of all NHLBI-supported articles published during the same period [36].

### Limitations of this study

In this study, we explored certain indicators of impact resulting from the published output from data re-use. It should be not conflated with the impact of data-sharing. Intention to share data may not result in effective data sharing, effective data sharing may not result in effective re-use, effective re-use may not result in a published output.

Importantly, it is worth noting that both citation metrics and Altmetric data are merely a surrogate for real research impact and interest; both scores have been identified as being correlated [37–41]. While the Altmetric Attention Score has been used in previous research [42] and seems to be an acceptable substitute for media attention and a relevant indicator for measuring interest and implication by the public in scientific research, it should obviously not be confused with scientific importance. Plus, Altmetric is highly gameable for some of their aspects such as tweets. It is however way more difficult to game with certain components such as guidelines. To provide a more comprehensive picture, we therefore described qualitatively how the different re-uses were cited by policy documents. Despite being increasingly used by journals, the construction of the Altmetric Attention Score with different weights attributed to altmetrics [43] is somewhat debatable and it is unknown how much this could impact our findings. It is uncertain whether the AAS information provided is complete. Similarly, its use as a composite criterion can create interpretation issues. Therefore we have detailed all its different dimensions one by one. The low coverage of all altmetrics, except for Twitter, was highlighted in a 2013 study [44]. It is possible that the Altmetric assessment might have missed some policy sources resulting in an underestimation of the true impact of these re-uses.

In addition, while we relied on an exhaustive review of various data repositories, there were still few re-uses included in our study. And indeed, while the repositories host a large volume of clinical trial data, they are still under-used with a small number of data requests in comparison to the volume of available data [20]. This could have resulted in limited power for our study, as we did not calculate the sample size prior to the study, making it difficult to detect a small difference between the 2 groups. In addition, any comparison between groups can be affected by confounders (e.g. research field, year of publication). We tried to control for these biases using a matched control group based on the same journal, but the matching criteria can be criticized, and unmeasured or residual confounders may still exist. Also, clinical trials were rare in the control group which could have an impact on the results, as it is likely that clinical trials attract more attention than other research designs, such as for instance observational studies. However this assumption does not seem to hold true in every field; in fact a review of the 50 most cited articles in the field of pediatrics showed that they were mainly observational studies [45]. On the other hand, a re-use of clinical trial data is not exactly the same as the primary use of this data. The results of the comparisons should therefore be interpreted cautiously and it is possible that the descriptive/qualitative analysis could be much more instructive than the comparison. Also finding an “unquestionable equivalent” for a secondary analysis, a methodological analysis or a re-analysis, which is not itself a re-use of clinical trial data, was near impossible. Even if an alternative matching would have been possible, the adoption of different definitions might have implied to select controls published much sooner or much later than the re-use. It means that any improvement in a sense may make matching worst in a second sense. It could have added much more, heterogeneity and, perhaps some confounding. It is indeed expected that the publication date can influence the amount of impact received. For example, matching an IPD meta-analysis with a meta-analysis on aggregated data in the same issue of a journal would have been quite unlikely. Confounding by time of publication, could also explain the negative correlation identified between primary papers and re-uses, as the importance of earlier papers could be underestimated by some social media (e.g. Twitter) that are relatively recent. Lastly, while a total of 89 re-uses of clinical trial data was identified, additional re-uses of data that did not result in publications are likely, meaning that the (somewhat low) impact of re-use described in this paper could be an overestimation compared with the true impact of data-sharing. Conversely it is also possible that some re-uses were not yet documented on the platforms at the time of the extraction and we did not include re-uses of clinical trial data that were not shared on the 3 data-sharing platforms. Data-sharing could also occur between teams, upon request, without the use of platforms.

### Conclusion

Data-sharing policies are coming progressively into effect with the aim of reforming the way clinical research is performed. However, since high-quality policies need to be evidence-based, it is necessary to assess whether they achieve the intended effects. In this perspective, and as a first step, this study documented the digital impact of published re-uses. We are however at an early stage in these policies and updating the data in the future with more knowledge and with additional altmetrics seems relevant, given the increasing proportion of re-uses [20]. In addition, adequate and reliable markers of impact and indeed usefulness [46] of these re-uses need to be developed, since neither altmetrics nor citation rates are a panacea for assessing impact.

Bearing in mind the numerous limitations of our study, we were not able to document a systematic impact of all re-uses in terms of media attention, nor in terms of use in policy documents. Still, the data collected in this study can inform future studies exploring the impact of clinical trial data-sharing. The need to modify some elements of the initial protocol may also feed the methodology of future research on this theme*.* This is an imperative to optimize current data-sharing practices.

## Supplementary Information

**Additional file 1.**

**Additional file 2.**

**Additional file 3.**

**Additional file 4.**

**Additional file 5.**

## Data Availability

The datasets generated and/or analyzed during the current study are available in the osf.io/fp62e repository.
