# Supplementary material for: Social media attention and citations of published outputs from re-use of clinical trial data: a matched comparison with articles published in the same journals
Source: BMC Med Res Methodol. 2021 Jun 6;21:119. doi: 10.1186/s12874-021-01311-z (PMC8182934; doi:10.1186/s12874-021-01311-z)
Supplement: Supplementary file 2 — Additional file 2. [file 12874_2021_1311_MOESM2_ESM.docx]

| **RE-USE** | **MATCH CONTROL** | **ICD CLASSIFICATION** |
| --- | --- | --- |
| Restoring Study 329: efficacy and harms of paroxetine and imipramine in treatment of major depression in adolescence | Mental illness, challenging behaviour, and psychotropic drug prescribing in people with intellectual disability: UK population based cohort study | 06 Mental, behavioural or neurodevelopmental disorders |
| Exploring changes over time and characteristics associated with data retrieval across individual participant data meta-analyses: systematic review | Development and validation of risk prediction model for venous thromboembolism in postpartum women: multinational cohort study | No proper ICD classification (methodology/statistics articles) |
| Antiepileptic drug monotherapy for epilepsy: a network meta-analysis of individual participant data | Botulinum toxin type A therapy for cervical dystonia | 08 Diseases of the nervous system |
| Adverse events following immunisation with four-component meningococcal serogroup B vaccine (4CMenB): interaction with co-administration of routine infant vaccines and risk of recurrence in European randomised controlled trials | Epidemiology of dengue and the effect of seasonal climate variation on its dynamics: a spatio-temporal descriptive analysis in the Chao-Shan area on China's southeastern coast | 01 Certain infectious or parasitic diseases |
| Mathematical modeling of HIV-1 transmission risk from condomless anal intercourse in HIV-infected MSM by the type of initial ART | Seasonality of influenza and its association with meteorological parameters in two cities of Pakistan: A time series analysis | 01 Certain infectious or parasitic diseases |
| Benefits and Harms of Sodium-Glucose Co-Transporter 2 Inhibitors in Patients with Type 2 Diabetes: A Systematic Review and Meta-Analysis | Effects of Liver Resection on Hepatic Short-Chain Fatty Acid Metabolism in Humans | 05 Endocrine, nutritional or metabolic diseases |
| Development and Validation of Machine Learning Models in Prediction of Remission in Patients With Moderate to Severe Crohn Disease | Performance of a Deep Learning Model vs Human Reviewers in Grading Endoscopic Disease Severity of Patients With Ulcerative Colitis | 13 Diseases of the digestive system |
| Representation of people with comorbidity and multimorbidity in clinical trials of novel drug therapies: an individual-level participant data analysis | Bleeding in cardiac patients prescribed antithrombotic drugs: electronic health record phenotyping algorithms, incidence, trends and prognosis | No proper ICD classification (drug-related articles) |
